# Address of Dr. Frank H. Hamilton

**Published:** 1878-03

**Authors:** 


### Address of Dr. Frank H. Hamilton.

Delivered at the Woman's Hospital, New York, November 22, 1877.

*Ladies and Gentlemen:*

Charitable associations, like clocks, need to be cleaned, oiled and wound up occasionally, or they would exhaust the forces under which they are driven forward, and sooner or later come to a dead stop. For this purpose, as I understand it, anniversaries are contrived.

During a whole year those connected with this Hospital have been employed in bringing in the sick, warming their cold feet, and wrapping their aching hearts with words of comfort and assurance; giving water to the thirsty, food to the hungry, wine to the fainting; answering to the calls of pain, and to the complaints of the peevish, and irritable, and restless, and of those who are unable to sleep, but only to think; and now, when the hands upon the dial indicate that twelve months have passed in this unceasing labor—ticking, ticking to the motion of the restless pendulum, from morning until night, and from night until morning, through 365 days, those who are in charge admonish us that the time has arrived when, in the ordinary course of events, the wheels would cease to move unless new forces were supplied.

To this end you, the officers and contributors, with others who are supposed to be interested, are called together to-day to listen to the reports, to examine into the condition of matters generally and in detail, and to suggest and supply the wants. You will notice whether anything is worn out or out of order; whether any oil is needed to prevênt friction, and then with united hands you will be called upon to wind up the main spring.

It might be well if Savings Banks and Life Insurance Companies were to imitate the example of charitable associations in this regard, and establish anniversaries for a similar purpose. If they were to do so it is probable it would not happen so often that they would need to be wound up by the courts.

In all machinery there is of necessity some friction; and the more complicated the machinery the greater will be the friction. In this Hospital, for example, there is the Board of Governors working together among themselves, the Board of Lady Managers working together among themselves, and the Board of Doctors working together in the same manner. Meanwhile each of these several boards are working with or against each other. There are the patients, also, the nurses and various other employés, constituting each a separate class, with more or less separate interests and opinions, perhaps. Each class grinding and pressing hard upon the other class, or leaning on each other for mutual support, like oxen under the yoke. To a certain extent, this pressing against each other is useful, as it tends to tighten and render compact the whole structure; and it renders the distribution of labor more equal. There can be no shirking where every one is crowded hard against his neighbor, and where every concussion is felt as in a solid body; but at the same time it causes dangerous friction, and hence the necessity for oil.

It was the original purpose of the founders of this Hospital to provide a place for women who were afflicted with certain maladies requiring for their relief the best surgical talents and skill, and also the most careful nursing—such only as experienced nurses could supply—and for those women only who had not the means to pay for such skill and service elsewhere, many of whom, therefore, had hitherto failed to obtain relief. This is still the great and benevolent purpose for which the Hospital is sustained from year to year, and constitutes its chief, if not its sole claim upon the charities of the public. If, up to the present time the original intention is not fully carried out, and a large portion of the building is given up to private and paying patients, the apology which must be offered is that hitherto its resources have been limited, and the paying patients have been admitted as a means of supplementing the resources, and of thus aiding in the support of the free beds.

In the few simple, and perhaps, wholly unnecessary, suggestions which I am about to make in relation to the working and management of this Hospital, and the character and conduct of its patients, this audience will understand that they have no intended reference or application to any except the free wards. That portion devoted to the paying patients is essentially a well appointed hotel, and so long as the guests have the means to pay they are not likely to suffer; when their pecuniary help is not needed none but the poor will be admitted.

The nurses, and all who attend upon the sick, ought to be kind and attentive.

This observation seems almost superfluous; but I am speaking now of the means to be employed to enable this, or any other Hospital of Charity to perform the humane intentions of its founders or supporters properly—to do the most good, and to pour the oil where it is most needed—and I naturally begin with the immediate attendants, because they are in the most close and constant contact with the sick, and so much of the comfort depends upon their intelligent and sympathetic administrations.

I am aware that the duties of this class are often very arduous and poorly paid, more so, perhaps, than the labors of any other class connected with a hospital; aud they must necessarily find themselves not unfrequently scarcely equal to the tasks imposed upon them; but then what may be properly required of them is. that they shall, at all times, control their own conduct, whether they are able to control the conduct of their patients or not. Under no circumstances ought an attendant be permitted to speak harshly or in passion to a sick person, especially if the patient is poor and dependent upon the charity of the Hospital. There is no need of this caution so far as the treatment of the rich is concerned. As I have before intimated, their money can always insure kind words; but the sick poor cannot buy either good services or kind words. They are, also, in consequence of their dependence, exceedingly sensitive and jealous of neglect.

There is, however, I may be permitted to say in this connection, one popular error in reference to these people, namely, that they are also "ungrateful." This is not my experience. On the contrary, as a rule, they are patient under suffering, and more grateful for services than the rich. The difficulty is, that we do not understand them; nor do they understand us, who would gladly do them a service. They are apt to think that, to a very great extent, all their mishaps and wrongs in life come from the avarice and selfishness of the rich, and that all the world is in league to oppress and rob them. This sentiment they carry everywhere with them, even into the hospitals of charity. They cannot be easily made to believe that they have any true friends, such as would do them an act of kindness or of benevolence without some selfish motive. They have not generally known, and it would surprise them to learn, that the doctor and you, good ladies and gentlemen, were not in some way paid for the services here given. But, if ever they become convinced, by your manner or by your unremitting kindness, that you are sincere in your efforts to serve them, their gratitude will be worth all the money and labor it has cost to secure it.

I have yet, after all my long experience in hospital life, to meet with a patient who could not be subdued and won by kindness; and, in my opinion, it ought to be an established rule, in all hospitals, that no attendant should ever address the patient in a tone of anger or of impatience, or of disrespect even, no matter how great the provocation.

It is because this is not always the established rule, and because, in many elemsoynary establishments the rule is quite the other way, that poor Mrs. Betty Higden had "run from it so many a year." The poor soul had probably had some brief experience at some time in her life in an alms-house or a Charity Hospital, and she knew that, although everything looked so sunny and green and pretty outside the great house, with its graveled walks and flowers and fountains, yet she had been chilled and almost frozen to death by the cold hearts inside.

"Its been chasing me all my life," said Betty, "but it shall never take me or mine alive;" and when pressed to take her precious little Johnny to the Hospital, she turned upon those whom she had just thought her friends like a wild animal at bay. "Ive done with ye. I would have fastened doors and windows and starved out 'fore I'd ever let you in. if I had known what you came for."

But catching sight of Mrs. Boffin's wholesome face, she suddenly relented, melting as under the influence of a sunbeam; and when the sweet, compassionate soul explained to her that she proposed to send little Johnny to a place where everybody did everything in their power to please and comfort and cure children, she asked with a gaze or wonder, "Is there then, really, such a place?" "Yes, Betty, on my word, and you shall see it." "You shall take him" (returned Betty, fervently kissing the comforting hand) "where you will, my deary." Mrs. Boffin had found the key to her heart and confidence very unexpectedly, and come what would Betty would never lose confidence in her again.

Ladies and gentlemen, this is pretty much always the way. The poor are are not so ungrateful as the world—I mean that portion of the world which is to-day uppermost—supposes them to be. When the world turns over, and those who are uppermost are undermost, the opinion of both classes may be changed.

My audience will excuse me, but I am very fond of reading Mr. Dickens' sermons, and there is in the briefer life of little Johnny another sermon, which it might be well to read, if our time would permit, although you have no doubt all read and wept over it before. It teaches that the sick, even the sick children in a charity hospital, may have their death beds made soft and their hearts made happy by those who attend upon them. And that they sympathize with each other, and often comfort and enrich themselves by pouring into each other's broken pitcher.

Those who minister at a sick bed, let me add, in continuation of my theme, should not wear an expression of solemnity nor of sadness; nor, on the other hand, should they indulge in unseemly levity; but they should carry always with them the oil of gladness and the refreshing light which always comes perpetually from a pleasant face.

Above all, means must be devised to prevent these unfortunate people from thinking. If only they could cease to think! Then they could sleep and eat, and the doctor's skill and anxious cares would be repaid by their speedy recovery. There is no medicine for the sick poor equal to forgetfulness of the past, of the present and of the future. Blessed is the man who has devised a cure for thinking.

Permit me to illustrate, by a single example, the value of forgetfulness as a means of curing purely physical maladies.

During one of those severe winters to which Buffalo is subject, and while in the performance of my regular duties at the Hospital of the Sisters of Charity in that city, I found one morning a little creature, composed of skin and bones, having three or four large starvation ulcers upon its face, lying upon its side in a corner bed, with its large blue eyes gazing steadily at the wall.

The sister in charge said that she had been left the day before by her mother, a very poor, industrious and temperate woman, but who for want of work, had been obliged to feed herself and her three children nearly all the winter on Indian corn meal, and of that they had very often not enough to appease their hunger. The parting with the mother had been painful and prolonged. The mother weeping, and the child striving with all its little remaining strength to maintain its grasp upon its mother's neck; and when at last the separation was effected and the mother was gone, the child sank as if from sheer exhaustion and despair, and permitted herself without opposition, to be undressed and put into bed, where, turning at once upon her side, she had lain all night with her eyes wide open and directed to the wall, shedding no tears, making no noise, and refusing obstinately to take either food, drink or medicine.

I do not think I then fully understood the case, but after I had left the hospital it came to me that probably she was thinking and that she needed diversion. My wife, to whom I related the incident, confirmed me in this opinion, and at her suggestion a doll with blonde hair was dressed with great judgment and exquisite taste, with plenty of blue, red and yellow ribbons, arranged in most violent and attractive contrasts.

Provided with this new medicine, which, I am sorry to say, is not mentioned in any of the works on materia medica, I visited the hospital early on the following morning. As I had anticipated, the condition of the child had not changed. During more than thirty-six hours she had lain in the same position, refusing to take anything which was offered to her, paying no attention to anything that was said to her, but gazing steadily at the dead wall. She was still thinking of her mother, of her little straw pallet, where she and her little sisters had lain huddled together to keep warm, of the bundle of stuffed rags which she used to nurse and pretend to feed, but which must now be starving, like herself, for want of food. She was thinking, probably of her dear home—as dear to her as yours is to you—from which she had been taken forcibly and placed in the hands of utter strangers.

It was my happy privilege to witness the result of our experiment with the new medicine. The doll with its blonde hair and its many colored ribbons and streamers was held between the great blue eyes and the wall, and after it had remained but a moment, one little skeleton hand came out slowly from under the cover, and the doll with its ribbons was drawn into the bed. We left her now to meditate upon, and become better acquainted with her splendid acquisition. When I returned from my walk through the wards, she had turned upon her back, and the doll was reposing upon her bosom, but not out of sight.

On the following day the mother—for the waif had now been fully adopted—was sitting up, supported by pillows, nursing the baby. She had slept well, and eaten more, the sister thought, than she ought to have eaten—less from a real sense of hunger, perhaps, than because her maternal instincts had taught her that she had now to provide nourishment for two instead of one.

From this day her recovery was rapid and complete.

If I have anything more to say on the matter of "oil,"—of the different qualities and uses of which I have spoken so much at length because of its great importance;—if I have anything more to say upon this subject, it is, that friction will be diminished very much in an institution like this, if every one attends strictly to his own business. The duties of all having been, as far as possible, carefully defined, let no man look to the right or to the left, but only forward, in the line of his own peculiar duties

This is the only way in which one can be sure of doing his own work well, and avoid obstructing the work of others. If any one is appointed to supervise and control the entire machinery, let him do it as well as he can; but I cannot conceive of any single person, being at the same time, a doctor, a financier, an architect, and a housekeeper.

Do not trouble yourselves especially about the peculiar ways in which others do their work. No two work alike, or think exactly alike in this world. Some are a bit too quick or fast, and some are a bit too slow; some are too forword, and others too backward; some too conceited, and others too humble; some too talkative, and some too reticent and secretive; some dress too much, and others too little; but it would be a sad state of human affairs if we were forbidden to enjoy at least one of these or other similar pet "infirmities," since most of us have two or more, natural or acquired, to be deprived of which would make us very uncomfortable, if not actually miserable.

Last of all, ladies and gentlemen, I am expected to call your attention to the main spring, called, in the language of commerce, "money." In this world, nothing moves without money. It is force! No matter how much oil you pour upon the wheels and axles, money is the main spring, and without it the machinery will stop. Money lifted every stone of these handsome buildings to its bed; and without money they would soon become empty ruins.

I believe it is always calculated that the main spring has length and strength enough to carry the Hospital beyond the twelve months; but to make sure for the year, it must be wound up now, and especially if you propose to increase the number of free beds, as I understand you do. I congratulate you, and all who are interested, upon that decision, for it is the only true foundation for a hospital to stand upon. Hospitals are seldom or never needed for any other purpose than to take care of the poor. If you expect to make this Hospital pay for all the trouble and money it has cost you, or for all that it may hereafter cost you, make every bed free, and fill it with poor people who cannot get help anywhere else, and there are plenty of them. Your beds will never be empty. We shall always be blessed with the poor; who will pay richly in gratitude, if you can only learn to touch the secret springs which unlock their deep and long pent-up reservoirs.

No one person is equal to the work now in hand, but at the main.spring, women generally show more strength and endurance than men. I have seen a woman who looked to me incapable of much physical effort, unlock the crooked and almost anchylosed fingers of a strong man, and take from his grasp a sum of money which he had no power of his own to relinquish, and no other man could have taken it from him. That was strange, was it not? Yet it is true; and I never saw the explanation of the trick, nor do I think the man did himself.

God bless you, ladies and gentlemen, for all you have done for suffering women in the erection and maintenance of this noble Hospital. It is one of the signs of progressive civilization when women are appreciated, and when they are made the special objects of our tenderest care. They constitute the moral leaven of this world. They are the most patient under suffering—the most dependent and helpless in misfortune of the two sexes; and if this Hospital is intended, on the part of my own sex, as a token of the debt of gratitude which we, and the world generally, owe to her, it ought to be large and free, and it should be maintained with a princely liberality.

